# A Case of Multiple Intracerebral Hemorrhages Due to Early-Onset Cerebral Amyloid Angiopathy With Alzheimer’s Disease: Neuropathological Changes Three Decades After Childhood Neurosurgery

**DOI:** 10.7759/cureus.77700

**Published:** 2025-01-20

**Authors:** Takumi Funakoshi, Minoru Yamada, Kazuna Ikeda, Kazuki Yokokawa, Taro Saito, Naotoshi Iwahara, Syuuichirou Suzuki, Yusuke Kimura, Yukinori Akiyama, Nobuhiro Mikuni, Shin Hisahara

**Affiliations:** 1 Department of Neurology, Sapporo Medical University, School of Medicine, Sapporo, JPN; 2 Department of Neurosurgery, Sapporo Medical University, School of Medicine, Sapporo, JPN

**Keywords:** amyloid beta, amyloid β, cerebral amyloid angiopathy, dura graft, early-onset cerebral amyloid angiopathy, neurosurgery in childhood

## Abstract

Cerebral amyloid angiopathy (CAA) is a disease in which amyloid beta (Aβ) is deposited in blood vessels and meninges in the brain. Cerebral amyloid angiopathy typically occurs in the elderly but is also known to occur in younger patients with a history of childhood head trauma or dura graft following neurosurgical procedures.

The patient was a 39-year-old female who had undergone neurosurgery for an arachnoid cyst in the right temporal lobe at the age of two years. Severe headache, dizziness, and right leg weakness developed abruptly. The Aβ_42/40 _ratio had decreased in cerebrospinal fluid. Brain MRI showed multiple cerebral hemorrhages. Considering CAA, a brain biopsy was performed. Pathological examination showed severe CAA in many leptomeningeal and cortical vessels.

We reported early-onset CAA after neurosurgery in childhood. In young patients with cerebral hemorrhages, it is necessary to consider early-onset CAA following childhood head trauma or neurosurgery.

## Introduction

Cerebral amyloid angiopathy (CAA) is a disease of amyloid beta (Aβ) deposition in blood vessels of the brain and meninges [1]. It has been suggested that CAA is associated with cerebral hemorrhage, inflammatory leukoencephalopathy, and cognitive impairment in the elderly. In recent years, cases of early-onset CAA have been reported in young patients who have undergone head trauma or neurosurgery in childhood. In particular, an association between cadaveric dura graft and the development of CAA has been strongly suggested [2]. In this report, we describe a case of early-onset CAA in which multiple cerebral hemorrhages occurred 37 years after neurosurgery, and brain biopsy revealed deposition of Aβ.

This article was previously posted to the Research Square preprint server on August 16, 2024.

## Case presentation

A 39-year-old female patient underwent craniotomy surgery for an arachnoid cyst in the right temporal lobe at the age of two years in 1984. It was not possible to ascertain whether a dural transplant was performed due to the absence of detailed surgical records. A ventriculoperitoneal shunt was inserted due to postoperative hydrocephalus. There were no episodes of blood transfusion in interviews with the patient's family. The patient developed symptomatic epilepsy and was started on antiepileptic drugs. Prior to admission, she had no cognitive impairment that interfered with her daily life. Six months before admission, an abnormal sensation appeared in the right lower extremity, resolving in about five minutes. Two months before admission, severe headaches and dizziness occurred suddenly and recurred repeatedly; she visited her previous hospital. Brain MRI showed multiple cerebral hemorrhages. The patient was admitted to our hospital to investigate the cause of the hemorrhage. 

**Figure 1:**
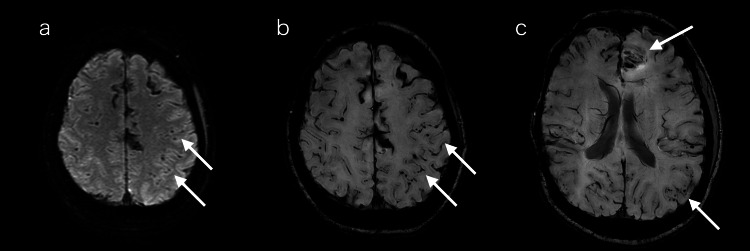
Brain MRI images on day one T2-weighted imaging showed multiple superficial hemosiderin depositions; white arrows indicated micro-bleedings. (a) Susceptibility-weighted imaging showed multilobed micro- and macro-hemorrhages and extensive superficial siderosis; (b, c) White arrows indicated hemorrhages and superficial siderosis.

The patient had aprosexia and mild cognitive impairment of 21/30 on the Mini-Mental State Examination (MMSE) (orientation to time: -1, calculation: -5, registration: -3) and mild muscle weakness in the right upper extremity. Blood tests showed no obvious abnormalities. Cerebrospinal fluid (CSF) examination showed a high total tau protein level of 1109 pg/mL (normal value is less than 410 pg/mL); the measurement of p-tau was not feasible due to the limitations imposed by the medical insurance coverage. The Aβ_42/40_ ratio had decreased to 0.11 (Aβ_40_: 2194 pg/mL, Aβ_42_: 251 pg/mL). The 14-3-3 protein and real-time quaking-induced conversion (RT-QuIC) were negative. Brain MRI showed hemosiderin deposition on the surface of the brain and numerous microbleeds in the subcortex on T2*-weighted imaging and susceptibility-weighted imaging (Figures 1a-1c). In contrast, there was no lobar hemorrhage. Electroencephalography demonstrated intermittent right temporal spikes and theta/delta activity enhanced by drowsiness and hyperventilation. A brain biopsy from the left frontal lobe was performed on day 27 to investigate the cause of multiple cerebral hemorrhages. 

**Figure 2:**
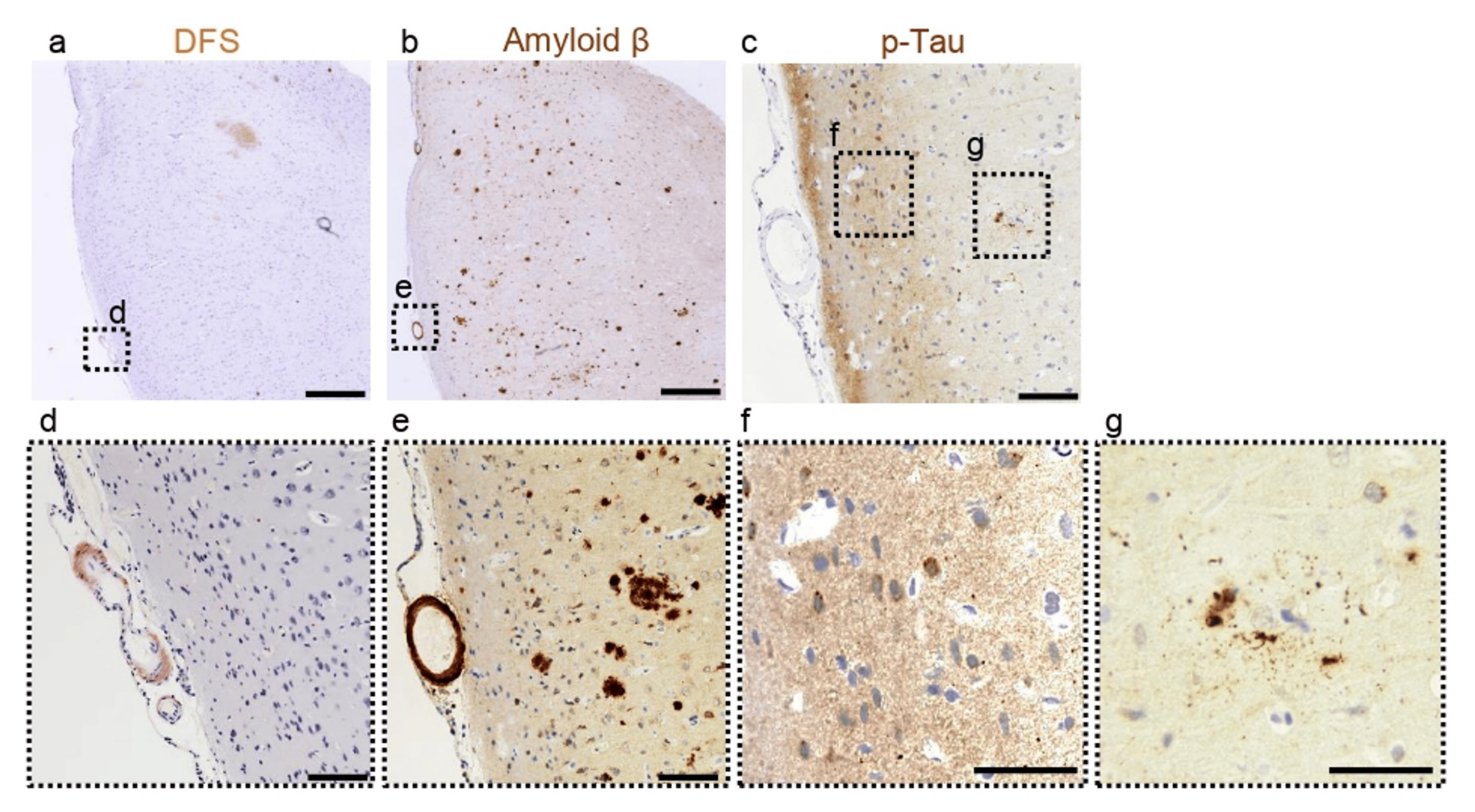
Pathological findings of the brain biopsy Amyloid staining using direct fast scarlet (DFS) showed the orange amyloid deposit on arterioles (a and d). Immunostaining for amyloid beta (Aβ) (6E10) indicated an Aβ-positive arterioles wall and senile plaques (b and e). Immunostaining for p-tau (AT8) showed pretangle in neurons and neuritic plaque around senile plaques (f and g). Scale bar: 1 mm (a and b), 200 μm (c, d, and e), and 100 μm (f and g).

Pathological investigation showed direct fast scarlet (DFS)-positive arterioles (Figures 2a, 2d) and Aβ deposition in the cerebral arterioles and cortex (Figures 2b, 2e). There was no Aβ deposition in the dura mater. The presence of p-tau (AT-8) in neurons, which may indicate the formation of pre-tangles, was observed. However, no neurofibrillary tangles were identified (Figures 2c, 2f). In addition, p-tau deposition was observed on neuritic plaques (Figures 2c, 2g). 

The patient was diagnosed with cerebral amyloid angiopathy-related hemorrhage. Testing for gene mutations causing Alzheimer's disease, such as APOE, APP, PSEN1, and PSEN2, could not be performed because the patient did not consent. We determined that the inflammatory findings associated with amyloid angiopathy were so mild in this case that treatment with glucocorticoids was not indicated. The patient was transferred on day 54 for rehabilitation.

## Discussion

We report an early-onset CAA case following neurosurgery in childhood. Cerebral amyloid angiopathy is a disease characterized by the deposition of Aβ in the meninges and the walls of small and medium-sized arteries in the brain. There are two types of CAA: type 1, in which Aβ is deposited in capillaries, and type 2, in which Aβ is deposited in small arteries [1]. Cerebral amyloid angiopathy is often seen in the elderly, and under the Boston criteria (version 2.0), probable CAA criteria are that the patient must be 50 years of age or older in cases where there are no pathological findings of CAA. And then, under Boston criteria (version 2.0), CAA can be diagnosed regardless of age if there are pathological findings of CAA [3]. We believe that our case falls into the category of probable CAA with supporting pathology. 

Recently, several cases of CAA-related cerebral hemorrhage occurring at the age of 49 years or younger, in which a dura mater graft was performed at an early age, have been reported and are referred to as early onset CAA [2, 4-7]. A comparable occurrence has been documented in patients with a medical history marked by neurosurgical interventions during childhood, excluding those who underwent dural transplantation [8]. Transmission of Aβ via dura mater graft, neurosurgical instruments contaminated with Aβ, and impaired postoperative Aβ clearance are thought to be the causes [8]. Alternative possibilities are a disturbance of the clearing system of cerebral Aβ, such as the glymphatic system and intramural periarterial drainage pathways [9]. 

Medical-induced Creutzfeldt-Jakob disease (CJD) has been observed in similar cases, and it is known that Aβ is transmitted via the same route as infectious prion protein (PrPSc) [8]. In 2008, Lyodura, a German-made cadaveric dried dura mater, was identified as a cause of medically induced CJD [10]. In Japan, about 20,000 people were transplanted with Lyodura every year from 1983 to 1987 [10]. The association between cadaveric dried dura mater transplants containing Lyodura and early-onset CAA is less frequent than that of medically-induced CJD, but a significant association has been suggested [11]. A previous report compared 23 cases of early-onset CAA-related hemorrhage with a history of neurosurgery or dura mater graft with 114 cases of medically induced CJD [10]. The mean time from surgery to disease onset was 11.6 years (±7.2) for medical-induced CJD and 34.5 years (±5.6) for early-onset CAA-related hemorrhage, suggesting a longer time for Aβ propagation compared to PrPSc [11]. In our case, the surgical record does not indicate whether the dura mater was implanted. However, the patient's mother remembers that her doctor told her that an artificial membrane was implanted, suggesting that a dura mater containing Lyodura was most likely implanted. 

The limitations of this case are that we were not able to assess cognitive function prior to the hemorrhage. The possibility that cognitive impairment was caused by cerebral hemorrhage or epilepsy cannot be ruled out. Furthermore, we did not test for Alzheimer's disease-related genes such as the APOE.

## Conclusions

We reported intracerebral hemorrhage due to early-onset CAA following neurosurgery in childhood. The patient had multiple microbleeds on MRI. In our case, the diagnosis of CAA was confirmed by the pathological results of Aβ and p-tau deposition on brain biopsy. Recent reports have shown an increase in the number of cases of CAA in young patients with a history of childhood head trauma or neurosurgery. An early-onset CAA is characterized by its onset decades after childhood head trauma or neurosurgery. In young patients with CAA, it is necessary to consider early-onset CAA following childhood head trauma or neurosurgery.
